# Exercise Recommendations and Practical Considerations for Asthma Management—An EAACI Position Paper

**DOI:** 10.1111/all.16573

**Published:** 2025-05-06

**Authors:** Oliver J. Price, Nikolaos G. Papadopoulos, Darío Antolín Amérigo, Vibeke Backer, Valérie Bougault, Stefano Del Giacco, Radoslaw Gawlik, Ibon Eguiluz‐Gracia, Enrico Heffler, Christer Janson, Vanessa M. McDonald, André Moreira, Andrew Simpson, Matteo Bonini

**Affiliations:** ^1^ School of Biomedical Sciences, Faculty of Biological Sciences University of Leeds Leeds UK; ^2^ Department of Respiratory Medicine Leeds Teaching Hospital NHS Trust Leeds UK; ^3^ Allergy Department, 2nd Pediatric Clinic National Kapodistrian University of Athens Athens Greece; ^4^ Lydia Becker Institute University of Manchester Manchester UK; ^5^ Allergy Department, Ramón y Cajal University Hospital Instituto Ramón y Cajal de Investigación Sanitaria (IRYCIS), Universidad de Alcalá Madrid Spain; ^6^ Department of Otorhinolaryngology, Head and Neck Surgery, and Audiology Rigshospitalet Copenhagen Denmark; ^7^ Université Côte d'Azur, LAMHESS Nice France; ^8^ Allergologia e Immunologia Clinica, Dipartimento di Scienze Mediche e Sanità Pubblica Università Degli Studi di Cagliari Cagliari Italy; ^9^ Silesian University School of Medicine Zabrze Poland; ^10^ Allergy Unit, Hospital Regional Universitario de Malaga, IBIMA‐Plataforma BIONAND RICORS Inflammatory Diseases Malaga Spain; ^11^ Personalized Medicine Asthma and Allergy–IRCCS Humanitas Research Hospital Rozzano Italy; ^12^ Department of Biomedical Sciences Humanitas University Rozzano Italy; ^13^ Department of Medical Sciences, Respiratory, Allergy and Sleep Research Uppsala University Uppsala Sweden; ^14^ School of Nursing and Midwifery The University of Newcastle Newcastle New South Wales Australia; ^15^ Department of Allergy and Clinical Immunology Centro Hospitalar Universitário de São João Porto Portugal; ^16^ EPIUnit–Institute of Public Health, Laboratory for Integrative and Translational Research in Population Health (ITR), University of Porto Porto Portugal; ^17^ Department of Pathology, Basic and Clinical Immunology Unit, Faculty of Medicine University of Porto Porto Portugal; ^18^ School of Sport, Exercise and Rehabilitation Sciences University of Hull Hull UK; ^19^ Department of Public Health and Infectious Diseases Sapienza University of Rome Rome Italy; ^20^ National Heart and Lung Institute (NHLI) Imperial College London London UK

**Keywords:** asthma, bronchoconstriction, exercise, management, physical activity, rehabilitation

## Abstract

Exercise is an important treatment for people with asthma and should be considered alongside pharmacological therapy when developing personalised asthma management plans. Despite this, there remains limited guidance concerning the practicalities of asthma‐specific exercise prescription. This European Academy of Allergy and Clinical Immunology task force was therefore established to achieve three fundamental aims: first, to provide an up‐to‐date perspective concerning the role of exercise for asthma management (i.e., describe the disease modifying potential of exercise and associated impact on asthma‐related extrapulmonary comorbidities); second, to develop pragmatic recommendations to facilitate safe and effective exercise prescription; and third, to identify key unmet needs and provide focused direction for future research. The position paper is structured as a practically focused document, with recommendations formulated according to best available scientific evidence and expert opinion, with an emphasis on providing healthcare providers with pragmatic advice that can be implemented during routine asthma review.

## Graphical abstract

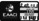

## 1 Introduction

Asthma is the most common chronic respiratory condition worldwide and a major source of economic and societal burden [1]. For the most part, pharmacological intervention forms the mainstay of treatment, with the Global Initiative for Asthma (GINA) recommending symptom‐driven low dose inhaled corticosteroid (ICS)‐formoterol as the preferred first line, with a step‐wise approach to maintenance treatment in those that continue to report high symptom burden [2]. Whilst this strategy has been shown to reduce the risk of exacerbations when compared to short‐acting beta‐2 agonists (SABA) prescribed as mono‐therapy [3], sub‐optimal disease control remains a long‐standing issue across all levels of asthma severity [4], and therefore other therapies with proven efficacy need to be considered when implementing personalised asthma management.

**FIGURE 1:**
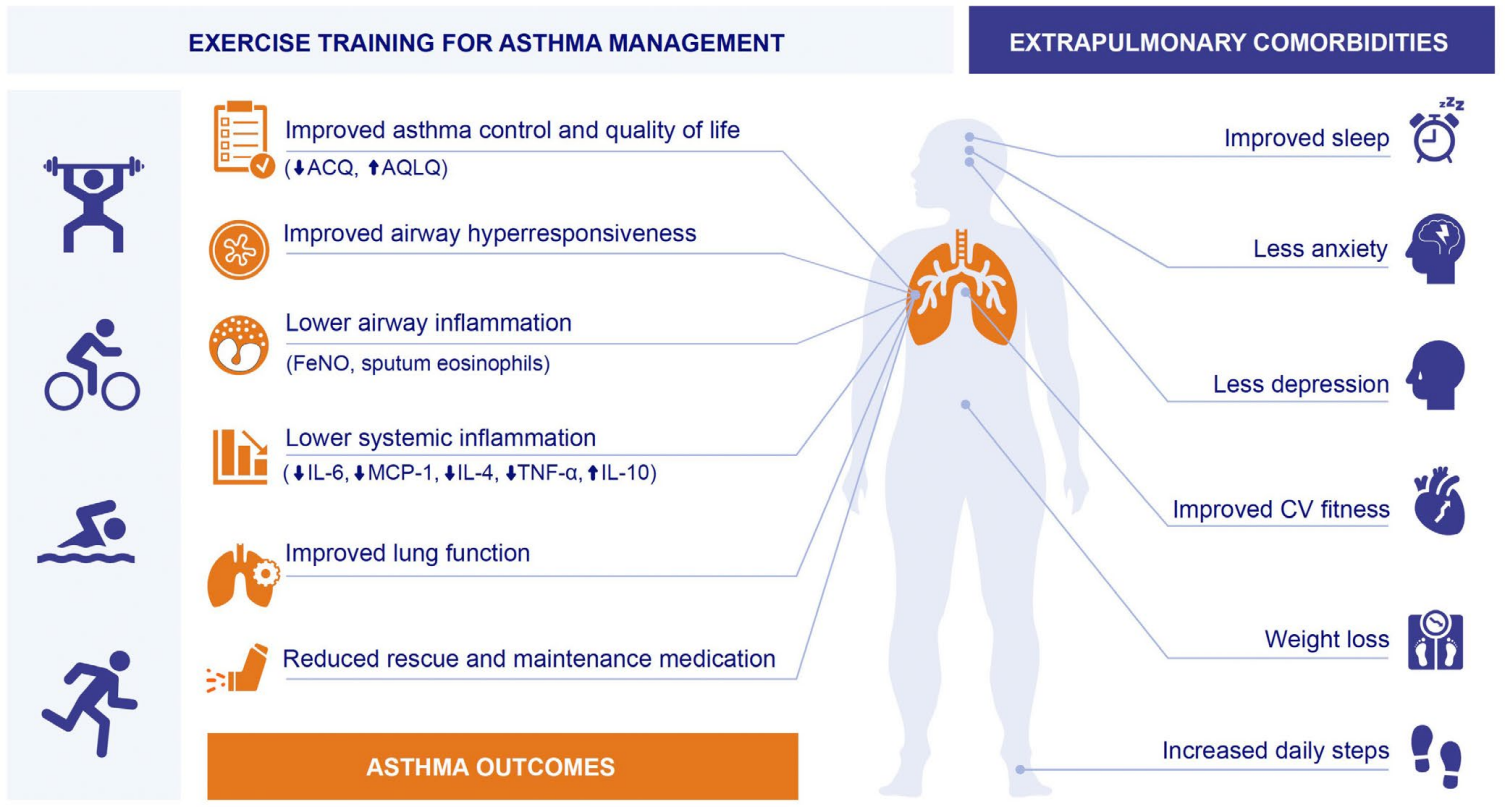
Impact of exercise training on asthma‐related outcomes and extrapulmonary comorbidities (summary of evidence from recent RCTs).

**FIGURE 2:**
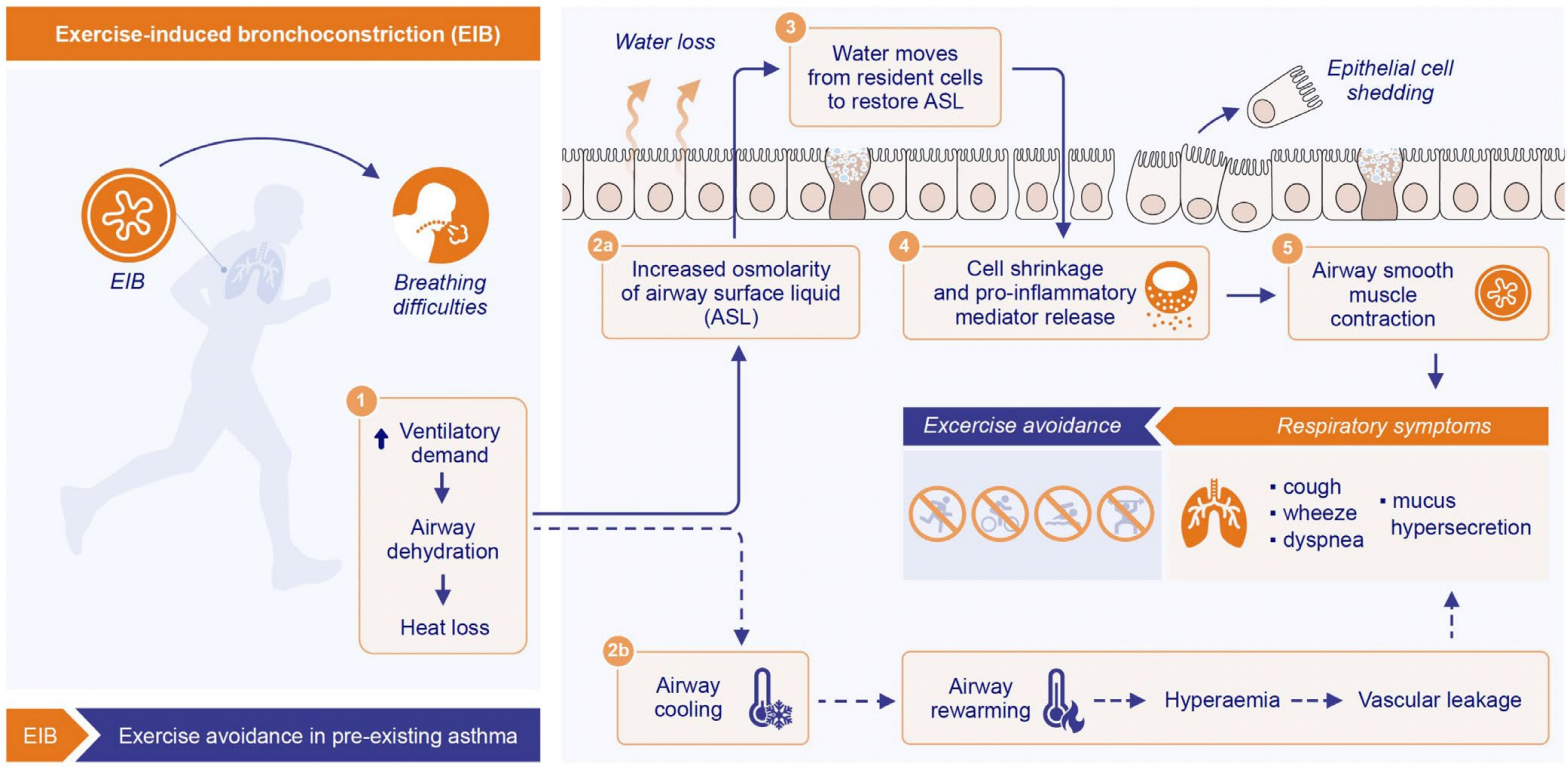
Exercise‐induced bronchoconstriction contributing to exercise avoidance in pre‐existing asthma.

In this respect, it is now apparent that engaging in regular moderate intensity aerobic exercise has the potential to modify disease via modulation of inflammatory and immune responses [5, 6, 7], and benefits a multitude of asthma‐related outcomes and extrapulmonary comorbidities (Figure 1). However, despite a growing body of evidence supporting the importance of exercise for asthma management [8, 9, 10, 11], it is typical for individuals with asthma to engage in lower levels of physical activity in comparison to healthy counterparts [11, 12]. The underpinning factors that contribute to physical inactivity in the general population are complex and multifaceted [13]; however, for individuals with asthma, it is thought to relate, at least in part, to disease specific barriers such as fear of provoking respiratory symptoms as a consequence of exercise‐induced bronchoconstriction (EIB) (i.e., transient lower airway narrowing [≥ 10% fall in FEV_1_]) that occurs during, or most often post exercise [14] (± ventilatory irregularity in the form of breathing pattern disorder), and/or severe exacerbation [15] (Figure 2).

Whilst the importance of exercise and physical activity in the context of asthma management has previously been described [16, 17], and endorsed in the current GINA report [2], there remains limited guidance concerning the practicalities of exercise prescription [18]. This European Academy of Allergy and Clinical Immunology (EAACI) task force was therefore established to achieve three fundamental aims: first, to provide an up‐to‐date perspective concerning the role of exercise for asthma management (i.e., describe the disease modifying potential of exercise and associated impact on asthma‐related extrapulmonary comorbidities); second, to develop pragmatic recommendations to facilitate safe and effective exercise prescription; and third, to identify key unmet needs and provide focused direction for future research.

## 2 Methodology

This task force was chaired by OJP with membership drawn from a multidisciplinary expert panel under the EAACI Research and Outreach Committee (ROC) [18] including allergists, clinical immunologists, pulmonologists, respiratory nurses, human physiologists and exercise scientists. Following initial discussion regarding the task force focus, original research (i.e., observational studies and randomised controlled trials [RCTs]) and systematic reviews and meta‐analyses published between 2014 and 2024 were identified in peer‐reviewed literature using the following search terms: ‘asthma’, ‘exercise‐induced asthma or bronchoconstriction’ or ‘airway hyper‐responsiveness [AHR]’ in combination with ‘exercise’ or ‘physical activity’ (i.e., conforming to the Scale for Assessment of Narrative Review Articles [SANRA] [19]). Pulmonary rehabilitation interventions or studies evaluating breathing exercises in isolation were excluded. The position paper was subsequently drafted by task force members according to clinical and research expertise, with an edited version approved by all authors prior to publication. Ten priority unanswered research questions were developed by task force members following group discussion and expert consensus (Box 1). The final position paper is structured as a practically focussed document, with recommendations formulated according to best available scientific evidence and expert opinion, with an emphasis on providing healthcare providers (e.g., physiotherapists, exercise physiologists, rehabilitation instructors etc.) with pragmatic advice that can be implemented during routine asthma review.

BOX 1 **Key unanswered research questions.**

**Table:** 

Identify key biological factors that drive the heterogeneous response to exercise training—and determine how this relates to asthma specific endotypes (i.e., comparing type II high vs. type II low)
2 To establish the optimal or most effective exercise ‘dose’ according to specific asthma severities and sub‐types and how this interacts with asthma control
3 In the obese asthma phenotype—does exercise modulate asthma‐related inflammation directly, or inflammation driven by adiposity?
4 Does exercise improve bone and skeletal muscle health in those exposed to long‐term ICS and oral corticosteroids therapy?
5 To determine the relationship between exercise and serious adverse events (i.e., exacerbation frequency/hospitalisation etc.)
6 How does exercise impact adherence to pharmacological treatments—and does this lead to a step‐down in therapy?
7 What is the impact of climatic conditions and air pollution on the ability to undertake exercise?
8 What is the effect of small but frequent doses of exercise on asthma outcomes and long‐term behaviour change/habit formation?
9 To determine the effect of asthma‐specific education and promotion interventions on long‐term exercise engagement
10 To utilise qualitative research methods to explore patient barriers and facilitators to exercise engagement and understand healthcare providers knowledge and experience of exercise prescription in the context of asthma management

## 3 Exercise for Asthma Management—What Do We Know?

### 3.1 Immunomodulatory and Anti‐Inflammatory Effects of Exercise

Asthma is a heterogeneous condition with respect to severity and sub‐type, with distinct patterns of airway inflammation reflecting diverse pathophysiological mechanisms (endotypes) that lead to variable clinical presentation (phenotypes) [20]. Type‐2‐high asthma is characterised by raised eosinophilia and is considered to be the main driver of allergic asthma, involving complex crosstalk between the airway epithelium and innate and adaptive immunity. Type‐2‐low asthma encompasses neutrophilic and paucigranulocytic asthma, whereas mixed granulocytic asthma is characterised by the co‐existence of eosinophilic and neutrophilic inflammation [21].

Exercise is recognised to effect immune function via various physiological mechanisms that contribute to alterations to innate and adaptive immunity [22]. In the acute setting, moderate‐to‐vigorous‐intensity exercise is associated with an increase in the synthesis and release of pro‐inflammatory cytokines (e.g., tumour necrosis factor alpha [TNF‐α], interleukin [IL]‐8, IL‐1β) [23], whereas in the chronic setting, exercise is thought to elicit an anti‐inflammatory effect, thus acting to reduce the development and progression of chronic disease [24]. The mechanisms that contribute to the anti‐inflammatory effects of regular moderate‐intensity exercise include: (i) lower visceral fat mass (i.e., reduction in pro‐inflammatory adipokines such as TNF‐α); (ii) increased production of anti‐inflammatory myokines (i.e., peptides or proteins secreted or released from contracting skeletal muscle); (iii) reduction in the expression of toll‐like receptors on monocytes and macrophages, with downstream inhibition of pro‐inflammatory cytokines [24].

To date, asthma‐related studies investigating the immunomodulatory and anti‐inflammatory effects of exercise have primarily focused on allergic and eosinophilic disease using models of ovalbumin‐sensitized mice [25, 26, 27, 28, 29]. Collectively, experimental animal studies provide strong evidence to suggest that regular aerobic exercise decreases airway inflammation, AHR, and structural airway remodeling via a reduction in the expression of T helper 2 (Th2) cytokines (IL‐4, IL‐5 and IL‐13) [25, 26, 27] and chemokines (e.g., monocyte chemoattractant protein [MCP‐1] and IL‐8), and an increase in the expression of anti‐inflammatory cytokines such as IL‐10 and IL‐1Ra [25, 27].

Despite this, there currently remains a limited number of high‐quality mechanistic studies to corroborate these findings in humans. Indeed, only one RCT has evaluated the impact of continuous aerobic exercise training on AHR (assessed via gold‐standard direct bronchial provocation methods) over the past decade [30]. In the landmark study by Pinto and colleagues, aerobic exercise (2× weekly 25‐min training sessions over a 12‐week period) was shown to improve AHR by one doubling dose of histamine and reduce serum IL‐6 and MCP‐1 in adults with moderate‐to‐severe asthma [30]. In addition, the exercise programme reduced sputum eosinophil count and fractional exhaled nitric oxide (FeNO) (an indirect biomarker of type 2 inflammation, signalling activation of IL‐4/IL‐13 pathway) in patients with raised airway inflammation (i.e., sputum eosinophils > 3% or FeNO > 26 ppb) [30]. In support of these findings, acute moderate intensity exercise has been shown to be associated with decreased FeNO [31] and reduced sputum eosinophil count [32]. Whilst further research is required, these data indicate that the therapeutic benefit of exercise are potentially endotype dependent, with greater anti‐inflammatory effects observed in response to moderate intensity exercise in those with type‐2‐high asthma.

### 3.2 Continuous Moderate‐To‐Vigorous‐Intensity Aerobic Exercise

To improve cardiorespiratory fitness and promote health and well‐being, it is recommended that adults undertake moderate intensity “steady state” aerobic exercise for a minimum of 30‐min five times per week, or vigorous‐intensity aerobic exercise for a minimum of 20‐min, three times per week (e.g., jogging, running, cycling, spinning, rowing, swimming etc.) [33]. Importantly, exercise is recognised to elicit comparable physiological adaptations in those with and without asthma, with studies in athletic cohorts indicating that individuals with moderate expiratory airflow limitation (i.e., FEV_1_ < 50% predicted) are still capable of achieving physical fitness levels (i.e., VO_2_max—defined as the maximal amount of oxygen an individual can consume) consistent with non‐asthmatic individuals with normal lung function [34].

The majority of asthma‐related exercise training studies have employed interventions in the moderate to vigorous‐intensity domain, with recent RCTs consistently reporting improvements in validated markers of asthma control and quality of life (assessed via the Asthma Control Questionnaire [ACQ] [35] and Asthma Quality of Life Questionnaire [AQLQ] [36]), psychosocial health, functional capacity and improved body composition (i.e., reduced adiposity and increased muscle mass in the context of the obese asthma phenotype) [30, 37, 38, 39, 40, 41, 42]. Whilst exercise training has previously been reported to have no effect on indices of resting lung function [17], the most recent systematic review and meta‐analysis of RCTs (k = 11 studies) (*n* = 543; 74.8% females) concluded that aerobic exercise (> 8 weeks duration) has the potential to improve asthma control and lung function, but has no clinically meaningful effect on markers of airway inflammation (i.e., FeNO and sputum eosinophilia) [43]. However, the findings from this analysis should be interpreted with caution, as the quality of evidence was graded as ‘low’ due to substantial heterogeneity and inconsistencies between studies (i.e., lack of standardisation concerning asthma sub‐types, disease severity, and intensity and duration of exercise training interventions). It should also be noted that a recent RCT observed no effect on spirometric indices of lung function following either moderate or vigorous‐intensity aerobic exercise [41]. RCTs evaluating continuous aerobic exercise training interventions are summarised in Table 1.

**TABLE 1:** Randomised controlled trials evaluating continuous moderate‐to‐vigorous‐intensity aerobic exercise training interventions.

First author (ref)	Year	Study population	Intervention	Outcome measures	Key findings
França‐Pinto [30]	2015	*n* = 43 adults with moderate or severe asthma 20–59 years	*Exercise training* (*n* = 22) 2× weekly 25‐min vigorous aerobic training sessions over 12‐weeks on indoor treadmill (+ sham intervention) *Sham* (*n* = 21) 2× weekly 30‐min supervised yoga breathing exercise programme over 12‐weeks	AHR (primary outcome), serum inflammatory cytokines, IgE, airway inflammation, clinical control (exacerbation, daily symptoms, ACQ and AQLQ), lung function and exercise capacity	Exercise improved AHR by 1 doubling dose of histamine and significantly reduced IL‐6 and MCP‐1 Exercise improved AQLQ and asthma exacerbation. No effect observed for IL‐5, IL‐8, IL‐10, sputum cellularity, FeNO or ACQ‐7 Exercise arm: within‐group significant differences were observed for ACQ‐6 in those with uncontrolled asthma and in those with sputum eosinophil > 3% and FeNO > 26 ppb
Freitas [37]	2017	*n* = 51 obese adults with moderate‐to‐severe asthma BMI ≥ 35–40 kg/m^2^ 30–60 years	*Weight loss programme* Hypocaloric diet counselling session led by nutritionist and psychologist incorporating behaviour change techniques *Weight loss* + *exercise training* (*n* = 26) 2× weekly aerobic (50%–75% VO_2peak_) + resistance training (50%–70% 1‐RM using major muscle groups) over 12‐weeks *Weight loss* + *sham* (*n* = 25) 2× weekly stretching + breathing exercise over 12‐weeks	ACQ (primary outcome) AQLQ, exercise capacity, physical activity, peripheral muscle strength, lung function, body composition, airway inflammation, serum inflammatory cytokines	Weight loss + exercise training significantly improved ACQ, exercise capacity and body composition Weight loss + exercise training significantly improved FEV_1_, FVC and expiratory reserve volume—but no other changes in lung function parameters observed FeNO, CCL2, IL‐4, IL‐6, TNF‐α and leptin were significantly lower following weight loss + exercise 25(OH)D, IL‐10 and serum adiponectin and leptin/adiponectin ratio significantly improved following weight loss + exercise
Freitas^a^ [38]	2018	—	—	Physical activity, sleep quality, anxiety and depression scores	Physical activity (daily step count) and asthma symptom–free days significantly increased following weight loss + exercise Weight loss + exercise significantly reduced depression scores and risk of developing obstructive sleep apnoea Sleep efficiency and latency significantly improved following weight loss + exercise
Jaakkola [39]	2019	*n* = 89 adults with mild‐to‐moderate asthma 16–65 years	*Exercise training* (*n* = 44) 3× weekly ≥ 30‐min aerobic exercise sessions (70%–80% HRmax), muscle training and stretching over 24‐weeks *Control* (*n* = 45) Usual care without exercise intervention	ACT (primary outcome), asthma‐related symptoms, PEF variability	Exercise significantly improved ACT and shortness of breath No significant difference in wheeze, cough or phlegm production PEF variability remained unchanged between groups
Evaristo [40]	2020	*n* = 54 adults with moderate‐to‐severe asthma 30–65 years	*Exercise training* (*n* = 29) 2× weekly 40‐min aerobic exercise on indoor treadmill (60% HRR) over 12‐weeks *Breathing exercise* (*n* = 25) 2× weekly 40‐min breathing exercises (designed to stimulate nasal and diaphragmatic breathing to increase expiratory time and regulate breathing) over 12‐weeks	ACQ‐6 (primary outcome), daily symptoms, airway inflammation, exercise capacity, AQLQ, HADS, HRQoL, physical activity	Exercise and breathing interventions had a similar effect on ACQ, psychological distress, asthma‐free symptom days, physical activity, and airway inflammation Exercise arm: 2.6 times more likely to experience clinical improvement at 3‐month follow‐up Exercise arm: longer‐lasting benefit and greater reduction in rescue medication use
Valkenborghs [41]	2024	*n* = 41 adults with physician diagnosed asthma and episodic symptoms 18–55 years	*Exercise training* (*n* = 12) 3× weekly 45‐mn moderate‐intensity aerobic exercise (55%–70% HRmax) for 12‐weeks *Exercise training* (*n* = 15) 3× weekly 30‐min vigorous‐intensity aerobic exercise (70%–90% HRmax) for 12‐weeks *Control* (*n* = 14) Usual care and maintain physical activity levels for 12‐weeks	AQLQ (primary outcome), ACQ, lung function, airway and systemic inflammation, exercise capacity, body composition	Moderate‐intensity exercise training resulted in a clinically and statistically significant improvement in AQLQ and ACQ relative to control Vigorous‐intensity exercise training had a statistically (but not clinically) significant improvement in AQLQ and ACQ relative to control Moderate‐intensity exercise training significantly reduced sputum macrophage and lymphocyte count relative to control Lower android fat mass, but not change in exercise capacity, was associated with improved AQLQ and reduced sputum IL‐6
Kim [42]	2024	*n* = 51 adults with moderate‐to‐severe asthma 18–60 years	*Aerobic* + *breathing exercise* (*n* = 26) 2× weekly 35‐min constant‐load exercise (60%–80%) + breathing exercises using Buteyko technique over 10‐weeks *Control* (*n* = 25) 2× weekly 35‐min constant‐load exercise (60%–80% HRmax) aerobic exercise + muscle‐stretching (sham intervention) over 10‐weeks	ACQ (primary outcome), AQLQ, HADS, sleep quality, hyperventilation, exercise capacity, lung function, physical activity, thoracoabdominal kinematics	Aerobic + breathing exercises did not improve asthma control or any other outcome measures in comparison to exercise training is isolation

Abbreviations: 1‐RM, 1‐repetition maximum; ACQ, Asthma Control Questionnaire; ACT, Asthma Control Test; AHR, airway hyperresponsiveness; AQLQ, Asthma Quality of Life Questionnaire; BMI, body mass index; CCL2, chemokine ligand 2; FeNO, fractional exhaled nitric oxide; FEV_1_, forced expiratory volume in 1 s; FVC, forced vital capacity; HRmax, maximal heart rate; HRR, heart rate reserve; IgE, Immunoglobulin E; IL‐10, Interleukin‐10; IL‐4, interleukin‐4; IL‐5, Interleukin‐5; IL‐6, Interleukin‐6; IL‐8, Interleukin‐8; MCP‐1, monocyte chemoattractant protein‐1; PEF, peak expiratory flow; TNF‐α, tumour necrosis factor‐alpha.

^a^ Study population and intervention from Freitas et al. [37].

### 3.3 High‐Intensity and Repeated Sprint Interval Training

High‐intensity interval training (HIIT) or repeated sprint interval training consists of repeated bouts of vigorous‐intensity exercise followed by lower intensity or passive recovery periods (generally between 4 and 8 sets). The workout typically totals 20–60 min in duration with exercise and recovery periods alternating between 5 s and 8 min depending on the specific protocol and patient tolerability. HIIT workouts can be tailored to medical or personal requirements and baseline aerobic fitness, including a diverse range of exercise modalities [33]. The potential benefits of HIIT include comparable or greater physiological adaptations (e.g., improved skeletal muscle metabolic control and cardiovascular function) when compared to continuous aerobic exercise in a shorter time period, and elevated post exercise metabolism known as “EPOC” (excess post‐exercise oxygen consumption), which may contribute to increased fat oxidation [44]. The benefits of HIIT are becoming increasingly recognised in healthy individuals [45] and in people with cardiometabolic and chronic obstructive pulmonary disease (COPD) [46, 47]. The potential application of HIIT in the context of asthma management includes improvements in perceived dyspnoea, anxiety, fatigue and reductions in ICS therapy without compromising asthma control [48, 49, 50, 51, 52, 53, 54, 55, 56].

The rationale to consider prescribing HIIT in people with asthma includes: (i) the inability or desire to undertake or sustain continuous moderate to vigorous‐intensity aerobic exercise to elicit significant physiological adaptations (i.e., increase oxidative capacity of locomotor muscle and expression of anabolic hormones to stimulate muscle fibre hypertrophy); (ii) superior to continuous exercise in improving exercise capacity while inducing lower dyspnoea sensations; (iii) a preferable training option for individuals unable to undertake and sustain exercise due to severe dyspnoea and those at risk of developing exercise‐induced arterial hypoxemia (defined as oxygen saturation [SpO_2_] 93%–95% [or 3%–4% below rest]); (iv) lower ventilatory requirement post intervention at the same relative sub‐maximal workload (i.e., increased inspiratory capacity, reduced dynamic hyperinflation and reduced dyspnoea) [47]. Whilst the potential risk of developing EIB in response to vigorous exercise is an important consideration, HIIT has been shown to be a safe, feasible, and well‐tolerated form of exercise in adults with asthma, irrespective of pre‐intervention disease control, airway inflammation, or AHR [57]. RCTs evaluating continuous HIIT interventions are summarised for reference in Table 2.

**TABLE 2:** Randomised controlled trials evaluating high‐intensity interval training interventions.

First author (ref)	Year	Study population	Intervention	Outcome measures	Key findings
Toennesen [48]	2018	*n* = 125 non‐obese adults with asthma 18–65 years	*Exercise* (*n* = 29) 3× weekly HIIT spinning sessions for 8‐weeks *Diet* (*n* = 33) High protein (25%–28% energy) and low GI (≤ 55) diet including anti‐inflammatory components (higher amounts of vegetables, fruit, nuts, lean meat, fish and seafood) for 8‐weeks *Exercise* + *diet* (*n* = 37) Exercise and diet intervention simultaneously for 8‐weeks *Control* (*n* = 38) Usual care and encouraged to maintain usual physical activity and diet	ACQ (primary outcome), AQLQ, airway and systemic inflammation, AHR, lung function, exercise capacity, body composition	Exercise + diet group significantly improved ACQ and AQLQ relative to control Exercise group and diet group did not improve ACQ or AQLQ compared with control VO_2_max significantly improved post intervention in the exercise group and exercise + diet group relative to control. No significant changes were observed in the diet or control groups All interventions resulted in a significant reduction in body weight and body fat mass relative to control No significant changes were observed in any groups post intervention for sputum cell counts, FEV_1_, FeNO or AHR
Bentzon^a^ [49]	2019	*n* = 60 non‐obese adults with asthma 18–65 years	1‐*year follow‐up* Exercising group: exercise alone combined with exercise + diet (*n* = 33) vs. non‐exercising group: diet alone combined with control (*n* = 27)	—	No significant difference in VO_2_max or ACQ (i.e., regressed to pre‐intervention values within 1‐year from study completion) between groups
da Silva [52]	2022	*n* = 55 adults with moderate‐to‐severe asthma	*Constant‐load exercise* (*n* = 27) 2× weekly 30‐min cycling at 70% maximal watts for 12‐weeks *HIIT cycling* (*n* = 28) 2× weekly 30‐min cycling start at 80% and increasing to 140% maximal watts (30‐s work: recovery ratio)	VO_2_peak (primary outcome), ACQ‐6, AQLQ, HADS, lung function, physical activity, airway and systemic inflammation	CLE and HIIT resulted in similar improvements in aerobic fitness and clinical improvements in psychosocial distress HIIT significantly lowered perceived dyspnoea and fatigue and resulted in significantly higher physical activity relative to CLE No significant difference in ACQ‐6 or lung function between interventions. HIIT resulted in a minimal clinically important difference in ACQ‐6 No change in markers of systemic or airway inflammation
Pitzner‐Fabricius [54]	2023	*n* = 129 adults with persistent asthma (defined as all year‐round symptoms) 18–75 years	*Exercise* (*n* = 88) 3× weekly 30‐min HIIT cycling at 90% HR_max_ (10–120 s with active low intensity recovery between intervals) over 6‐months *Control* (*n* = 41) Usual lifestyle and physical activity habits	ICS therapy reduced by 25% of baseline dose at 6‐months (primary outcome), ICS use, ACQ‐5, AQLQ, rescue inhaler use, AHR, airway and systemic inflammation, lung function, exercise capacity, physical activity	Individuals who reduced their ICS dose by 25% or greater at 6‐months was higher in the exercise group ICS use reduced in favour of the exercise group without compromising asthma control and sustained at 12‐month follow‐up Exercise resulted in a significant reduction in weight loss and fat percentage and improvement in VO_2_max and physical activity No significant difference observed for FeNO, blood eosinophil count, FEV_1_, or AHR

Abbreviations: ACQ, Asthma Control Questionnaire; AHR, airway hyperresponsiveness; AQLQ, Asthma Quality of Life Questionnaire; CLE, constant‐load exercise; FeNO, fractional exhaled nitric oxide; FEV_1_, forced expiratory volume in 1 s; HADS, Hospital Anxiety Depression Scale; HIIT, high‐intensity interval training; HRmax, maximum heart rate; ICS, inhaled corticosteroid; VO_2_max, maximal oxygen uptake; VO_2_peak, peak oxygen uptake.

^a^ 1‐year follow‐up from Toennesen et al. [48].

### 3.4 Impact of Exercise on Asthma‐Related Extrapulmonary Comorbidities

Extrapulmonary comorbidities such as obesity, anxiety, depression, gastroesophageal reflux disease and cardiometabolic disease are common in people with asthma, particularly those with moderate‐to‐severe disease [58]. Extrapulmonary comorbidities are often considered ‘syndemic’ – where biological interactions occur as two or more disease states, which adversely interact, and negatively influence the trajectory of each condition [59]. In the context of asthma management, if comorbid illness is identified early, exercise has the potential to improve disease control and benefit extrapulmonary outcomes [60]. In the past, exercise training studies in people with asthma have primarily focused on functional capacity and/or physical activity status as primary outcome measures [55, 61, 62, 63]. However, recent studies have also reported improvements in a variety of extrapulmonary comorbidities (Figure 1). For example, exercise combined with dietary restriction has been shown to elicit a significant reduction in adiposity compared to exercise training in isolation in overweight and obese asthma [64]. Similarly, alongside improvements in body mass index (BMI), symptom scores and step‐based activity, combined aerobic and resistance exercise + weight lost interventions have been shown to improve depression, sleep efficiency and latency and lower the risk of obstructive sleep apnoea in adults with coexisting obesity and asthma [38].

Other potential clinically relevant asthma‐related extrapulmonary comorbidities include impaired bone health (i.e., osteopenia/osteoporosis and fragility fractures) as a consequence of long‐term ICS and oral corticosteroid therapy [65]. Similarly, accumulating evidence suggests that adults with moderate‐to‐severe and/or refractory asthma are at increased susceptibility to skeletal muscle weakness (akin to observations in COPD [66]), and that patients with uncontrolled asthma have increased fat mass and decreased muscle mass [67]. The prevention of skeletal muscle dysfunction and decreased mass (i.e., sarcopenia) is important due to the strong association with functional capacity and overall quality of life [67]. For this reason, resistance training and flexibility exercises (alongside aerobic exercise training) that target all major muscle groups are currently recommended at least twice per week for people with asthma to maintain strength and mobility [33]. In addition, impaired core muscle function is associated with worse breathing symptoms in severe asthma [68], suggesting that interventions such as yoga and/or Pilates (i.e., exercise specifically targeting core strength, balance, posture, stabilisation and flexibility) should be employed alongside aerobic and resistance‐based exercise [69]. Similarly, inspiratory muscle training using pressure threshold loading is recognised to enhance inspiratory muscle strength and therefore may offer value as an adjunct therapy to improve core function in adults with asthma [70, 71].

## 4 Practical Approach to Exercise Prescription

### 4.1 Initial Asthma Review and Follow‐Up Consultation

The initial assessment of an individual presenting with typical asthma symptoms should prompt a standard asthma review in accordance with international guidelines, including a detailed medical history in combination with spirometry and bronchodilator reversibility testing [2]. The diagnostic work‐up can also include allergy testing (i.e., demonstrating sensitisation via positive skin prick test and quantification of serum immunoglobulin E [IgE]), and in cases where the diagnosis remains unclear (i.e., normal resting lung function and negative bronchodilator reversibility) a direct bronchial provocation test (i.e., methacholine or histamine challenge) is required to evaluate AHR [72]. FeNO and/or blood eosinophil count can be utilised to support asthma phenotyping and follow‐up assessment to monitor the response to ICS therapy [73].

Individuals that report exercise‐related symptoms should be referred for a form of indirect bronchial provocation test (i.e., exercise challenge, eucapnic voluntary hyperpnoea or inhaled mannitol) to confirm or refute evidence of EIB [14]. The pharmacological approach to the prevention and management of EIB includes daily maintenance treatment (depending on underlying asthma severity) or anti‐inflammatory relievers (i.e., ICS‐formoterol or ICS‐SABA) as needed when bronchoconstriction is exclusively elicited by exercise [2, 74]. In patients that continue to report a high symptom burden despite treatment escalation, it is important to consider the differential diagnosis or common comorbidities (notably, exercise‐induced laryngeal obstruction and/or breathing pattern disorder) due to the potential impact on pulmonary mechanics and symptom perception. For a detailed overview of the diagnosis and management of exercise‐related allergic and respiratory disorders (i.e., conditions that often co‐exist in people with asthma) see Price et al. [14]. In those with a confirmed diagnosis, a personalised asthma management plan involving a continuous cycle (i.e., assess, adjust treatment and review response) should be developed and tailored according to individual patient needs [2].

In terms of exercise promotion, as a starting point, an assessment of current activity status should be obtained. Whilst questionnaires such as the International Physical Activity Questionnaire Short Form (IPAQ‐SF) provide a convenient and cost‐effective method to quantify physical activity, there remains concern regarding potential under‐and overestimation when relying on patient self‐report in isolation on an individual basis [75]. Due to the exponential rise in the availability and use of mobile technologies with in‐built activity sensors (with the functionality to monitor daily step‐count), modern smartphones provide an opportunity to retrospectively review objective physical activity data (with appropriate consent obtained) during consultation [76].

Incorporating open‐ended questions should be encouraged to understand barriers and facilitators to exercise engagement and physical capacity to lead an active lifestyle (e.g., “how do you feel about your current activity status and/or ability to undertake exercise?”) [15, 77, 78], and in turn, develop personalised recommendations to optimise enjoyment and promote long‐term adherence. Implementing established behaviour change strategies (e.g., goal setting, action planning, positive reinforcement and motivational techniques etc.) should also be utilised [79], alongside pragmatic advice to increase activities of daily living (e.g., use the stairs rather than escalator at work to increase daily step‐count etc.). For those with limited experience of exercise training, it is important to provide reassurance that an increased perception of effort and mild sensation of breathlessness is a physiologically appropriate and expected response to moderate to vigorous‐intensity exercise [80]. Likewise, delayed onset muscle soreness (DOMS) 24–48 h post exercise is a normal and expected response to vigorous exertion, particularly for untrained individuals or following unaccustomed exercise, and resolves spontaneously within 5–7 days. Finally, educating patients concerning the benefits and relatively low risks of undertaking exercise is imperative given very few asthma deaths in conjunction with exercise or sports participation have been reported over the past three decades [81].

### 4.2 Assessing Exercise Tolerance and Functional Capacity

Exercise testing is employed in clinical practice to evaluate exercise tolerance and functional capacity [82] (defined as the ability to undertake activities of daily living that require sustained, submaximal aerobic metabolism [83]) but also has application in the context of exercise prescription to permit tailored recommendations at an appropriate intensity or workload that is well tolerated by patients [33]. Cardiopulmonary exercise testing (CPET) is considered the gold‐standard method to evaluate exercise tolerance as it provides a comprehensive integrated assessment of the cardiac, respiratory, and musculoskeletal systems under physiological stress [82, 84]. CPET provides breath‐by‐breath data on respiratory gas exchange, including minute ventilation (V_E_), oxygen uptake (VO_2_), carbon dioxide production (VCO_2_), breathing frequency, and tidal volume (V_T_), as well as hemodynamic parameters (such as blood pressure and SpO_2_) and subjective assessment of dyspnoea via the Borg CR10 scale (0–10 category‐ratio scale for breathlessness intensity rating) [85]. CPET also has application in the assessment of erratic or irregular breathing in response to incremental exercise in those with suspected breathing pattern disorder [86].

CPET is typically performed on an electrically braked cycle ergometer or a treadmill, although portable metabolic devices permit exercise testing in alternative and sport‐specific settings. While cycle ergometry is considered the preferred method of testing (due to lower relative risk of falls, reduced motion artefact and potential for arterial blood sampling), treadmill testing is considered advantageous on the basis that walking and running are considered more familiar activities to most patients [82, 87]. CPET can be performed incrementally or at a constant work rate. Incremental protocols involve a gradual increase in workload over 10–12 min in a continuous or stepwise mode. Exercise is usually continued until volitional fatigue, unless medical complications occur (e.g., angina, ischaemia, uncontrolled hypertension, marked oxygen desaturation, dizziness or mental confusion) resulting in early termination. Incremental protocols can be used to evaluate exercise performance and provide insight concerning potential mechanisms contributing to exercise intolerance and exertional dyspnoea.

A typical protocol for constant work rate testing includes a 1‐3‐min warm‐up, followed by an abrupt increase in workload to approximately 75%–80% maximal work rate. In those susceptible to EIB, or with marked or fixed airflow obstruction, inhaled SABA may be indicated to optimize the assessment of cardiopulmonary capacity [33]. The constant work test is generally continued to a symptom‐limited maximum and is primarily used to monitor disease evolution and to determine the response to therapeutic intervention [88]. The potential application of CPET in the assessment and management of people with asthma (i.e., evaluating the cause of exercise intolerance, presence of dynamic hyperinflation and perception of exertional dyspnoea) has recently been described [89].

The 6 min walking test (6MWT) is a surrogate test used to evaluate functional capacity in people with asthma [90, 91, 92, 93]. 6MWT measures the distance walked on a hard flat surface over 6 min while monitoring heart rate and SpO_2_. The strongest clinical indication for performing a 6MWT is as a one‐time measure of functional status, but also to evaluate the response to intervention. The 6MWT does not determine peak oxygen uptake, diagnose the cause of dyspnoea on exertion, or evaluate the mechanisms of exercise intolerance and therefore, the information should be considered complementary rather than a direct replacement for CPET [94].

### 4.3 Implementing Established Exercise Training Principles

Investment in resources including staffing and facilities is essential to facilitate safe and effective exercise prescription at scale. From a practical pointofview, the design of an exercise programme should be tailored according to individual needs with the aim of improving physical fitness and general health [33]. Due to the relatively low number of high‐quality RCTs, asthma‐specific evidence‐based guidelines are not currently available; therefore, exercise training principles for healthy adults (adjusted according to patient capabilities) are currently recommended for people with asthma [33]. The general approach to exercise prescription consists of four core components: frequency, intensity, time, and type (modality)—which is often referred to as the “FITT principle” or “FITT‐VP principle” when factoring training volume and progression [33]. The FITT principle represents the exercise ‘dose’ or quantity required to improve health and well‐being. While several methods can be used to calculate exercise intensity, heart rate dependent methods (e.g., % heart rate max) are susceptible to over‐and‐underestimation [95]; therefore, heart rate reserve (HRR) or VO_2_ reserve (VO_2_R) (calculated based on CPET derived data) are considered the most appropriate for deconditioned individuals, older populations, and those with chronic disease [33]. This recommendation is particularly pertinent for individuals with asthma given the potential effects of acute beta‐2 agonist administration on systemic vascular function [96]. An alternative and more simplistic approach to guide exercise intensity involves the Borg rating [6, 7, 8, 9, 10, 11, 12, 13, 14, 15, 16, 17, 18, 19, 20] of perceived exertion RPE (scale of 6 [rest] to 20 [maximum effort]) based on a subjective assessment of physical sensations and subjective experiences (i.e., increased heart rate, breathing frequency and perception of effort/fatigue) (Table 3).

**TABLE 3:** Exercise recommendations and practical considerations for asthma management.

Exercise intensity^a^	Aerobic		Resistance
	%HRR or VO_2_R	RPE (6–20)	%1‐RM
Very light	< 30	< 9 (very light)	< 30
Light	30–39	9–11 (very light to fairly light)	30–49
Moderate	40–59	12–13 (fairly light to somewhat hard)	50–69
Vigorous	60–89	14–17 (somewhat hard to very hard)	70–84
Near maximal to maximal	≥ 90	≥ 18 (very hard)	≥ 85
*Training zone calculations* ^a^			
HRR method	HR_max_ = 220 − age Target HR = [(HR_max_ − HR_rest_) × % intensity] + HR_rest_		
VO_2_R method	Target VO_2_R = [(VO_2max/peak_ − VO_2rest_) × % intensity] + VO_2rest_		
*Exercise prescription* ^a^	Aerobic	Resistance	Flexibility
Frequency	3–5 days per week	2–3 days per week Prevent muscle wasting and promote bone health in those regularly using inhaled and oral steroids [65]	≥ 2–3 days per week (daily recommended)
Intensity	Moderate intensity (40%–59% HRR or VO_2_R) with progression to vigorous intensity domain (including HIIT) if well‐tolerated Example HIIT protocol: 80%–95% HRR or VO_2_R followed by lower intensity recovery periods (40%–50% HRR or VO_2_R) For patients that routinely monitor PEF—use measurements to adjust exercise intensity/training programme	Strength: 60%–70% of 1‐RM for beginners; ≥ 80% for more experienced individuals Endurance: < 50% for 1‐RM	Whole body stretching to tolerance
Time	30–40 min per day	Strength: 2–4 sets of 8–12 repetitions Endurance: < 2 sets of 15–20 repetitions	Static stretching for 10–30 s; 2–4 repetitions
Type (modality)	Aerobic exercise using large muscle groups such as walking, running, cycling and swimming Non‐weight bearing exercise recommended for those with raised BMI or musculoskeletal conditions Swimming—warm humid environment limits desiccating airway stimulus to induce EIB Non‐chlorinated pool recommended to limit exposure to chloramines/prevent exacerbations Hydrostatic pressure of water may act to strengthen/condition respiratory muscles Apnoea (breath‐holding) may improve control of breathing	Weight machines or body weight exercise for beginners; incorporate the use of free weights for more experienced individuals Inspiratory muscle training—pressure threshold loading used to strengthen diaphragm and accessory respiratory muscles Consider as an adjunct to aerobic and resistance‐based exercise in motivated patients 30‐breathes twice per day (intensity 50% PImax) with progressive overload	Yoga and Pilates (static and dynamic stretching that targets large muscle groups)
*Practical considerations*			
Medical clearance	Inactive or sedentary individuals and those with signs or symptoms suggestive of comorbid cardiovascular, metabolic of renal disease undergo medical evaluation prior to beginning an exercise programme Avoid exercise following acute exacerbation until symptoms and lung function have improved		
Warm‐up	5–10 min light to moderate intensity activities Incorporate high‐intensity intervals in those with EIB to promote refractory period 5–10 min whole body stretching		
Medication	Adhere to personalised asthma management plan [2] Daily maintenance treatment or anti‐inflammatory relievers (i.e., ICS‐formoterol or ICS‐SABA) as‐needed when bronchoconstriction is exclusively elicited by exercise [2, 74]		
Safety considerations	Rescue medication on‐person (or in close proximity) at all times Terminate exercise bout if symptoms persist or become problematic		
Exercise session	Exercise intensity can be monitored using heart‐rate dependent training zones or RPE according to perceived effort ‘Talk test’ should be considered—i.e., ability to talk but not sing during moderate‐intensity exercise and struggle to speak in short sentences during vigorous‐intensity exercise Wearable activity monitors and fitness tracking apps should be considered to support remote assessment with real‐time feedback Exercise volume can be increased with progression achieved over time by adjusting frequency, intensity and time		
Cool‐down	5–10 min light to moderate intensity activities 5–10 min whole body stretching		

Abbreviations: 1‐RM, 1‐repetition maximum; EIB, exercise‐induced bronchoconstriction; HIIT, high‐intensity interval training; HR, heart rate; HR_max_, maximum heart rate; HRR, heart rate reserve; ICS, inhaled corticosteroid; PEF, peak expiratory flow; PImax, maximum inspiratory pressure; RPE, rating of perceived exertion; SABA, short‐acting beta‐2 agonist; VO_2max/peak_, maximum/peak oxygen uptake; VO_2_R, oxygen uptake reserve; VO_2rest_, resting oxygen uptake.

^a^ Adapted from Liguori et al. [33].

**FIGURE 3:**
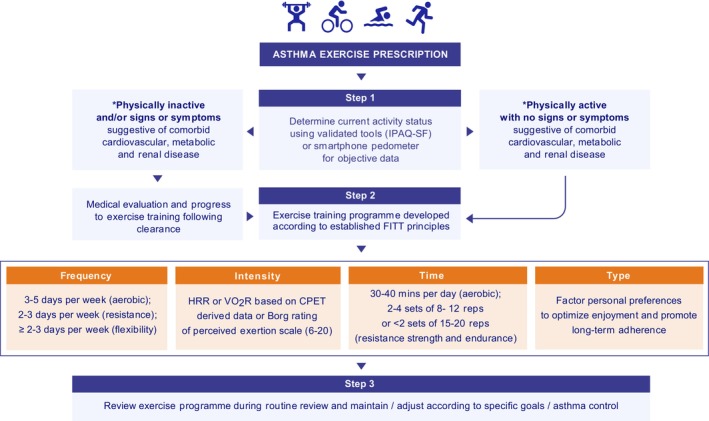
Flowchart summarising approach to exercise prescription in people with asthma (*physically active defined as undertaking a minimum of 30‐min aerobic exercise five times per week, or vigorous‐intensity aerobic exercise for a minimum of 20‐min, three times per week).

In advance of undertaking an exercise programme, it is important that inactive individuals (i.e., those that do not to undertake planned, structured physical activity of at least 30‐min duration at moderate intensity on at least 3‐days per week) or sedentary individuals (i.e., sitting, reclining or lying down for prolonged periods) and those with signs or symptoms suggestive of comorbid cardiovascular, metabolic, or renal disease undergo medical evaluation [97, 98, 99]. Irrespective of the requirement for medical clearance, exercise should be prescribed at an appropriate workload according to baseline activity status or cardiorespiratory fitness, with consideration for other potentially relevant barriers (e.g., non‐weight bearing exercise such as cycling or swimming may be preferable in patients with raised BMI or musculoskeletal conditions etc.). From a safety point of view, rescue medications should be on person (or in close proximity) at all times. A structured warm‐up should also be encouraged to promote a refractory period—an approach shown to be effective in some individuals with asthma due to mast cell mediator depletion, thus acting to protect against EIB [100]. In those that continue to report persistent or troublesome exercise‐related symptoms, it is important to review and adjust the individual's personalised asthma management in accordance with GINA guidance [2] (Figure 3).

### 4.4 Environmental Factors and Considerations

The environment in which exercise is undertaken is an important consideration for people with asthma on the basis that V_E_ in excess of 30 L/min results in a shift in breathing pattern from almost exclusive nasal airflow to combined oral and nasal airflow [101]. As a result, the lower airways are exposed to a greater quantity of unconditioned air and potential deposition of airborne allergens and other inhaled noxious particles during moderate to vigorous‐intensity exercise [102]. Environmental factors that can trigger or worsen asthma symptoms include changes in meteorological conditions, the composition of inhaled air, which may contain high concentrations of chemical components (e.g., trichloramines in chlorinated swimming pools), air pollutants (i.e., various types and sizes of particulate matter [PM], ozone [O_3_], nitrogen dioxide [NO_2_], sulphur dioxide SO_2_), and airborne allergens [103]. The atmospheric temperature is also relevant given increased exposure to dry cold air is a common cause of EIB in a significant proportion of people with asthma [104]. Whilst inhalation of warm humid air is typically considered to be less ‘asthmogenic’ [105], breathing hot air has been shown to increase airway resistance via the cholinergic reflex pathway and potential activation of airway C‐fibres (i.e., acting to provoke bronchoconstriction and cough) [106].

Of particular concern for individuals with asthma engaging in exercise is the ongoing climate change crisis as the pollen season is projected to occur earlier in the year and for longer durations, with heatwaves, landscape fires, and episodic air pollution also expected to occur more frequently. It is generally considered that the benefits of regular exercise outweigh the health risks associated with air pollution in the general population [107]; however, individuals with asthma are at risk of EIB or exacerbation [108] and therefore strategies need to be considered on an individual basis and incorporated as part of a personalised asthma management plan to negate the potential adverse effects of environmental exposures during exercise [109, 110] (Table 4).

**TABLE 4:** Asthma‐specific strategies to mitigate the potential adverse effects of environmental exposures during exercise.

Strategy	Practical considerations	
*Monitor environment*	Review daily updates on current and forecast air quality, weather and pollen count from trusted sources (e.g., local government websites, apps) and adjust exercising environment according to air quality recommendations	
*Media alerts*	Subscribe and follow public health recommendations for susceptible individuals in case of wildfires, PM, ozone, heatwave, pollen or thunderstorms	
*Minimise exposure*	*Location*	Select green or blue spaces to undertake physical activities. Exercise away from main roads, airports, harbours and other sources of pollutants such as industrial facilities. Select travel routes that minimise exposure to air pollution Indoor activity (± HEPA filter) may be an option in extreme temperatures or high air pollution, especially if the two are combined or persist for several days or months Ozone levels are generally lower indoors, especially if the room is air conditioned and the windows are closed. Indoor exercise is therefore advisable during an O_3_ alert. Ventilation should be sufficient to remove CO_2_ from the room, and sports facilities with portable air cleaners and high‐efficiency particulate air filters are recommended In winter months, care should be taken to avoid moisture and dust mites, mould, bacteria, viruses, and fungi. In summer months, air conditioning units can dry the air and increase risk of EIB If available, altitude therapy during symptomatic episodes should be considered to synergically reduce exposure to heat, pollution and pollen, especially in people with severe or poorly controlled asthma [111]
	*Time*	Air quality is generally less polluted early in the morning
	*Facemasks*	Respirators (facemasks) considered on an individual basis factoring the potential benefits (reduced exposure) vs. adverse effects (increased work of breathing/perceived dyspnoea). Facemask with specific filters (N95, FFP2 or FFP3) may be partly effective against air pollution characterised by high PM levels Heat and moisture exchanging mask (or scarf or snood) can attenuate the airway response (protect against EIB and cough) during cold and dry air inhalation [112]
	*Exercise modality*	Exercise intensity can be reduced if necessary or desired. Low‐ventilation activities such as resistance‐based exercise, balance activities, stretching, yoga, tai‐chi should be considered Exercise duration outdoors can be reduced and replaced with regular short bouts of indoor exercise (e.g., mobility training, short stair climbing, walking at home, light household activities etc.)
*Medication*		Adhere to personalised asthma management plan [2]
*Dietary supplements*		Limited evidence concerning the role of antioxidants, vitamins or omega‐3 PUFA supplementation on lung function or respiratory symptoms when exercising in a polluted environment Dietary supplementation should be considered in those with specific nutritional deficiencies

Abbreviations: CO_2_, carbon dioxide; EIB, exercise‐induced bronchoconstriction; HEPA, high‐efficiency particulate air; O_3_, ozone; PM, particulate matter; PUFA, polyunsaturated fatty acids.

### 4.5 Technology in Exercise Monitoring and Promotion

The approach to promoting health enhancing behaviours has traditionally focused on providing advice and support during medical consultation. However, the development and widespread availability of modern technology (i.e., wearable activity monitors, smartphones with in‐built pedometers and fitness tracking apps) provide an opportunity for remote assessment with real‐time feedback—thus supporting self‐regulatory and goal‐directed behaviour [79]. To date, there currently remains a limited number of studies specifically evaluating medical grade wearable activity monitors (i.e., accelerometers and pedometers) in people with asthma [113]. However, a recent umbrella review (*k* = 39 systematic reviews and meta‐analyses; *n* = 163,992) concluded that wearable activity monitors lead to a clinically important and sustained improvement in physical activity in a variety of clinical populations and age groups (> 1800 extra steps per day and 40‐min more daily walking) [114]. In addition, a separate meta‐analysis highlighted that wearable activity monitors in conjunction with established behaviour change techniques lead to the greatest improvement in step‐based physical activity in people with chronic airways disease [113].

Smartphone apps, fitness bands, and smartwatches provide automated and continuous monitoring of health and fitness parameters with reasonable accuracy; however, how this translates to long‐term exercise engagement has yet to be established [115]. Despite this, the best available evidence suggests that exercise interventions involving mobile apps or trackers with automated and continuous self‐monitoring and feedback (particularly those including text‐messaging and personalised features) lead to a significant improvement in step‐based physical activity in healthy adults [116]. Smartphone technology has the functionality to monitor atmospheric conditions (i.e., temperature, humidity, pollen count and pollution) and can therefore support the decision‐making process to mitigate against relevant asthma triggers and environmental exposures [117]. Heart rate monitors and smartwatches also permit the assessment of acute physiological responses at rest and in response to activity and therefore have application in the context of structured exercise training programmes (i.e., provide real‐time feedback to ensure appropriate heart rate‐dependent training zones etc.).

## 5 Exercise for Asthma Management—What Do We Need to Know?

### 5.1 Endotype‐Driven Approach to Exercise Prescription

Exercise training intervention studies conducted over the past decade have consistently demonstrated improvements in a variety of asthma‐related outcomes and extrapulmonary comorbidities [30, 37, 38, 39, 40, 41, 42, 48, 52, 54]; however, inter‐study variation exists in relation to the impact on exhaled and systematic inflammatory biomarkers, lung function, and AHR (Tables 1 and 2). The reason for the observed disparity remains unclear, but likely relates to asthma heterogeneity and the specific nature of the exercise intervention employed. Other relevant factors include: (i) demographic age, sex, BMI, and ethnicity, (ii) variation in clinical tools and outcome measures, (iii) interaction with pharmacological agents, and (v) physical activity, functional capacity, and asthma control at study entry. The latter is an important consideration on the basis that trained or physically active individuals with well‐controlled asthma (i.e., ACQ score < 0.75) are intuitively less likely to show a significant or clinically meaningful effect in response to exercise in comparison to inactive or sedentary individuals with uncontrolled asthma (i.e., ACQ score > 1.5). It is therefore important to account for potentially confounding variables in the design of future studies and interpretation of findings. In the same way, an endotype‐driven approach is recommended to tailor asthma management and predict response to pharmacological therapies [118]; stratifying the response to exercise training interventions in well‐characterised cohorts will help to elucidate underpinning mechanisms according to specific asthma sub‐types.

### 5.2 Exercise Dose–Response Relationship

The biological gradient (i.e., dose–response relationship) between exercise volume and asthma control remains to be fully established. Whilst the beneficial effects of exercise for people with asthma is convincing, it is important to acknowledge that prolonged vigorous‐intensity aerobic exercise (i.e., training volume exceeding 10–15 h per week), particularly when performed in allergy or irritant‐laden environments, can cause injury to the airway epithelium [119]. This concern is substantiated on the basis that one in four elite‐level endurance athletes have evidence of asthma and/or EIB [120], and that transient AHR is temporally associated with regular exercise, which appears to be reversible on training cessation [121]. To determine the optimal or most effective ‘dose’—further longitudinal population‐based research is therefore required to establish how exercise and physical activity ‘interacts’ with validated markers of asthma control and established clinical endpoints. For example, the application of non‐effort dependent methods of assessing small airway function via impulse oscillometry would offer particular value beyond conventional spirometric indices to determine the impact of exercise training on lung function and AHR [122, 123]. Likewise, non‐invasive imaging techniques (e.g., hyperpolarised magnetic resonance imaging) and exhaled breath analytics should also be considered to provide mechanistic insight.

### 5.3 Factors Contributing to Exercise Avoidance and Adherence

Identifying factors that contribute to exercise avoidance remains a priority to permit the development of targeted behaviour change strategies to promote long‐term adherence [15]. It remains apparent that the therapeutic effects of exercise are often short‐lived, with improvements in asthma‐related outcomes often regressing to pre‐intervention values within one year [49]. To address this issue, it is important that research is undertaken to understand key barriers, facilitators, and determinants of exercise avoidance and engagement (with consideration for general physical activity status) to permit personalised exercise prescription. Whilst the application of smart technology offers a promising platform for this purpose (i.e., monitoring and promoting exercise and physical activity), further research is required to confirm the efficacy of this approach in a real‐world setting.

### 5.4 Summary and Future Research Plans

Exercise is an important treatment for people with asthma and should be considered alongside pharmacological therapy when developing and reviewing personalised asthma management plans. Whilst the disease‐modifying potential of exercise for asthma management is biologically plausible, further high‐quality studies with adequate statistical power are required to elucidate underpinning immunomodulatory and anti‐inflammatory mechanisms according to specific exercise training interventions (with consideration for established FITT principles). To achieve this objective, we propose the development of an international task force and/or research consortium to facilitate knowledge exchange between clinicians and scientists and provide a platform to deliver multicentre research trials (employing consistent protocols and clinical endpoints) with long‐term follow‐up/surveillance. It is envisaged that adopting this approach will facilitate scientific progression over the next 5–10 years (i.e., shed light on key unanswered research questions [Box 1])—and ultimately support the long‐term ambition of developing evidence‐based, objectively graded, asthma‐specific exercise prescription guidelines.

## Author Contributions

O.J.P. chaired the task force and drafted early versions of the manuscript with expert contributions from all co‐authors (N.G.P., D.A.A., Vibeke Backer, Valérie Bougault, S.D.G., R.G., I.E.G., E.H., C.J., V.M.M., A.M., A.S., M.B.) according to clinical and research expertise. All authors provided approval of the final version of this manuscript to be published.

## Disclosure

O.J.P. confirms responsibility for the content of the manuscript on behalf of all EAACI task force members.

## Conflicts of Interest

O.J.P. reports grants from Merck and AstraZeneca outside the submitted work. D.A.A. reports grants from the Spanish Society of Allergology and Clinical Immunology (SEAIC), consulting fees from ALK‐Abelló, AstraZeneca, Chiesi, and Gebro, and speaker fees from AstraZeneca, Chiesi, Gebro, GlaxoSmithKline, Leti Pharma, Menarini, Novartis, Roxall, and Sanofi, all outside the submitted work. S.D.G. reports grants, advisory board, and speaker fees from AstraZeneca, Chiesi, GSK, CSL‐Behring, Novartis, Sanofi, and Takeda, outside the submitted work. V.M.M. reports grants from GlaxoSmithKline and advisory board and speaker fees from GlaxoSmithKline, Menarini, and Boehringer Ingelheim outside the submitted work. M.B. reports grants, advisory board, and speaker fees from AstraZeneca, Chiesi, Grifols, GlaxoSmithKline, Lallemand, Lusofarmaco, Menarini, Omron, and Sanofi outside the submitted work. N.G.P., Vibeke Backer, Valérie Bougault, R.G., I.E.G., E.H., C.J., A.M., and A.S. have declare no conflicts of interest.

## Data Availability

Data sharing not applicable to this article as no datasets were generated or analysed during the current study.
